# Upper cervical anterior fusion to C2 with temporary infrahyoid muscle detachment: a clinical case series and description of surgical technique

**DOI:** 10.1186/s13018-023-03937-9

**Published:** 2023-06-28

**Authors:** Naoki Okamoto, Rentaro Okazaki, Seiichi Azuma

**Affiliations:** 1 Department of Orthopaedic Surgery, Japanese Red Cross Saitama Hospital, 1-5 Shintoshin, Chuo-ku, Saitama, 330-0081 Japan; 2 Department of Orthopaedic Surgery, Yaizu City Hospital, 1000 Doubara, Yaizu-city Shizuoka, 425-0055 Japan

**Keywords:** Anterior cervical fusion, C2, Superior laryngeal nerve, Dyspnea, Dysphagia

## Abstract

**Background:**

Anterior cervical spine surgery to C2 (ACSS-C2) is a challenging procedure that often results in postoperative persistent dysphagia or dyspnea due to injury to the internal branch of the superior laryngeal nerve (iSLN) or the relatively narrow and soft oropharynx. This study aimed to describe the surgical outcomes of our modified approach with temporary infrahyoid muscle detachment during ACSS-C2.

**Methods:**

Patients who underwent ACSS-C2 at two institutions between June 2015 and January 2022 were prospectively enrolled. Intraoperatively, we performed temporary detachment of the infrahyoid muscle from the hyoid bone to improve laryngeal mobility and accessibility to C2. This procedure also allowed for the easy identification and preservation of the iSLN. We retrospectively investigated the surgery-related complications and outcomes of bony fusion.

**Results:**

Twelve patients were enrolled in this study; five and seven patients underwent single- and multi-level fusion surgery, respectively. Intraoperative preservation of the iSLN and proper visualization of C2 were achieved in all cases. Subsequent decompression and instrumentation were successfully performed. Two older patients (78 and 81 years) who underwent multi-level fusion experienced transient postoperative dysphagia. None of the patients required unplanned reintubation or revision surgery because of instrumentation failure. Solid bony fusion was achieved in all cases.

**Conclusions:**

Our modified approach with temporary infrahyoid muscle detachment during ACSS-C2 reduces the incidence of postoperative persistent dysphagia and dyspnea. However, in older patients at high risk for postoperative dysphagia, multi-level fusion should be avoided, and alternative procedures should be considered.

## Introduction

Anterior cervical spine surgery to C2 (ACSS-C2) is a challenging procedure that often results in postoperative dysphagia or dyspnea due to neurovascular injury of the specific regional anatomy around the upper cervical levels [1–3]. The internal branch of the superior laryngeal nerve (iSLN), which courses at the C3–4 level and provides innervation to the laryngeal mucosa, can be injured or accidentally ligated during dissection, which may lead to postoperative persistent swallowing dysfunction [4, 5]. The oropharynx, which is located at the C2–3 to C3–4 levels, is a relatively soft and narrow tissue that is not protected by skeletal structures [6]. Surgical exposure of C2 by placement of blade retractors could cause retropharyngeal edema, physical airway stenosis, and consequent respiratory failure [3, 7]. To the best of our knowledge, few studies have described the methods for anterior access to C2, with even less information describing the efficacy and risks of ACSS-C2 especially in cases of multi-level fusion. The aim of this study was to describe our modified approach for ACSS-C2 and investigate its surgical outcomes and complications.

## Methods

### Patient population

Written informed consent was obtained from all patients involved in this study. Data of patients who underwent ACSS-C2 at two institutions with a minimum follow-up of 12 months were prospectively collected from June 2015 to January 2022 and retrospectively reviewed.

We collected the patients’ baseline and surgical procedural characteristics, including age at the time of surgery, sex, preoperative diagnosis, preoperative symptoms, follow-up period (last visit or telephone interview), previous surgery, fused vertebral levels, type of surgical procedure, operation time, and estimated blood loss. We also investigated surgery-related complications and bone fusion outcomes.

### Surgery-related complications

We reviewed the surgery-related perioperative complications. Distinct postoperative dysphagia was defined as a condition requiring nasogastric tube feeding due to difficulty swallowing food after 1 week postoperatively. Dyspnea was defined as a condition requiring unplanned reintubation due to upper airway obstruction within 1-week postoperatively.

### Fusion outcomes

Bony fusion was evaluated on computed tomography scans obtained > 6 months postoperatively. To determine whether complete fusion was achieved, the continuity of the trabeculae on the sagittal and/or coronal view was examined independently by two surgeons.

### Surgical techniques

**Fig. 1:**
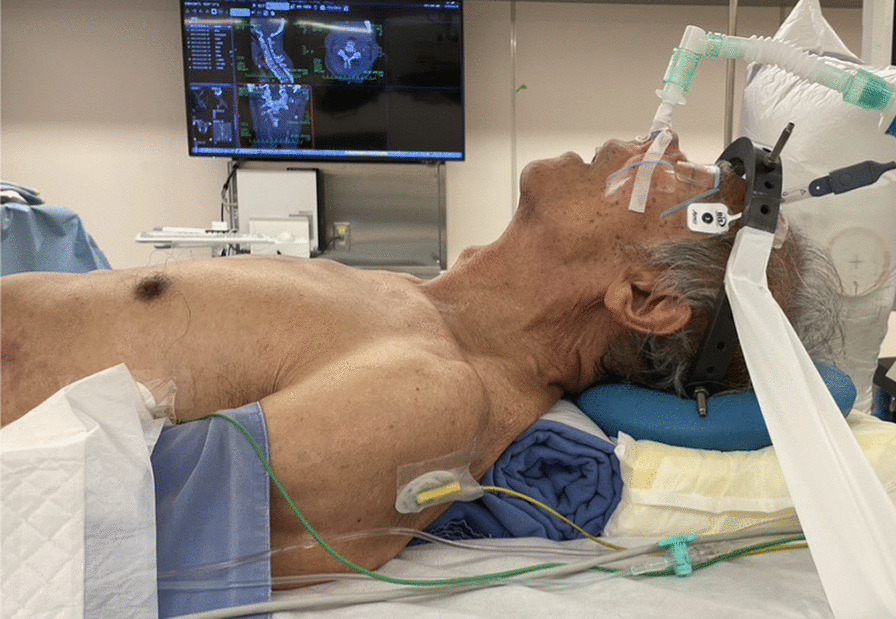
The patient was placed in a supine position with a shoulder roll to facilitate cervical extension and mandibular elevation. Nasal intubation was performed to allow intraoperative full-jaw closure and easy access to the upper cervical spine

**Fig. 2:**
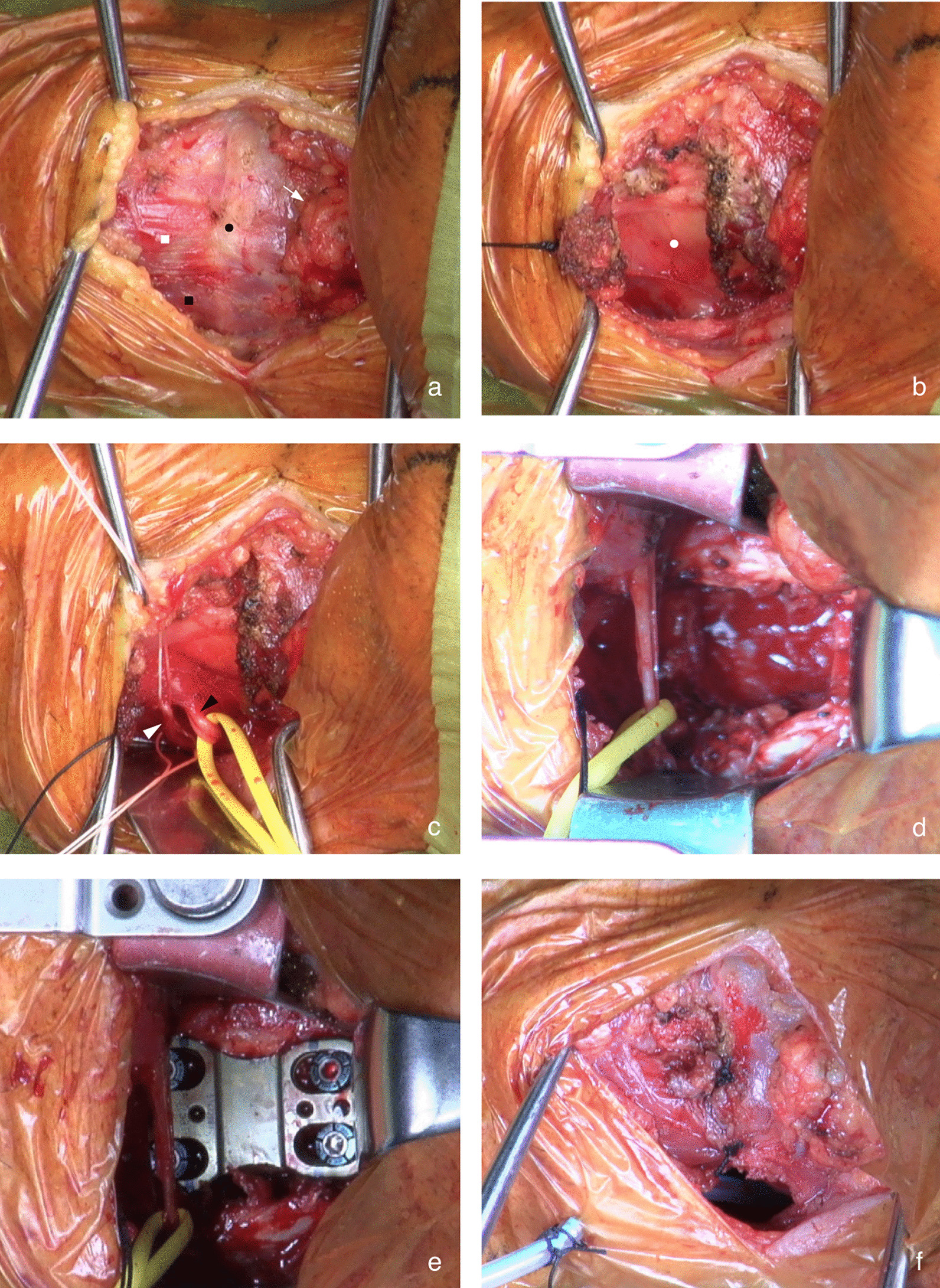
The surgeon’s view of left-sided transverse incision approach for C2–4 anterior corpectomy and fusion is shown. **a** After subplatysmal dissection, the hyoid bone (*black dot*), omohyoid muscle (*black square*), sternohyoid muscle (*white square*), and submandibular gland (*white arrow*) become visible. **b** Detachment of the omohyoid and sternohyoid muscles from the hyoid bone reveals the thyrohyoid membrane (*white dot*), which is pierced by the internal branch of the superior laryngeal nerve (iSLN). **c** The iSLN (*black arrowhead*) and superior laryngeal artery (*white arrowhead*) are identified and tagged via blunt dissection along the lateral edge of the larynx. **d** C3 corpectomy is achieved in the normal procedure. **e** The iSLN is preserved after C2–4 plate fixation. **f** The omohyoid and sternohyoid muscles are repaired and sutured to the hyoid bone before wound closure

**Fig. 3:**
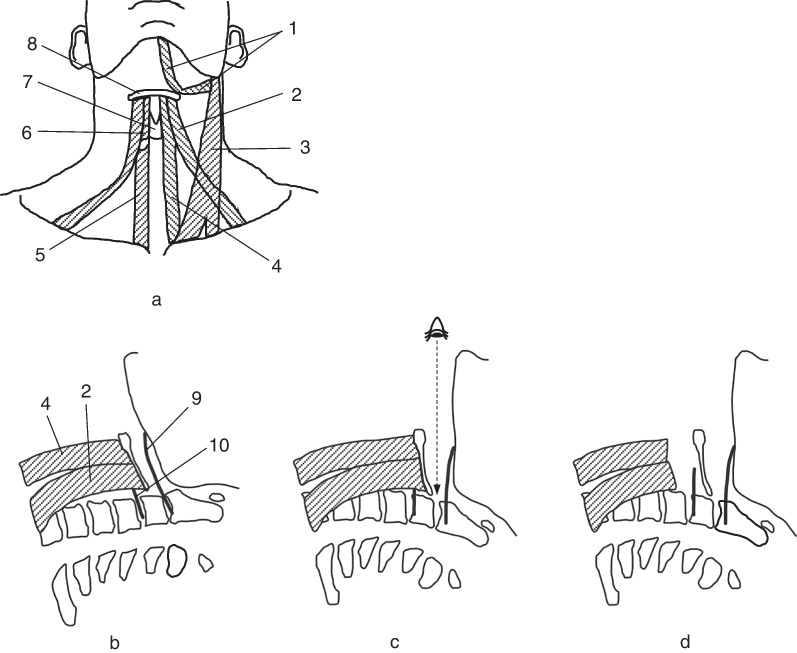
(1) Digastric muscle; (2) omohyoid; (3) sternocleidomastoid; (4) sternohyoid; (5) sternothyroid; (6) thyrohyoid; (7) thyroid cartilage; (8) hyoid bone; (9) hypoglossal nerve; (10) internal branch of the superior laryngeal nerve (iSLN). **a** Schematic image of the infrahyoid muscles is shown. **b** The mandible usually overlies the C2/3 disk in a neutral cervical position. The hypoglossal nerve and iSLN traverse the operative field at the C2/3 and C3/4 levels, respectively. **c** Cervical extension and chin-up position reduce mandible interference and enable perpendicular anterior access to C2; however, this position elevates the hyoid bone, resulting in increased infrahyoid muscles tightness and decreased laryngeal mobility. **d** Infrahyoid muscle detachment from the hyoid bone increases laryngeal mobility and reveals the thyrohyoid membrane, which is pierced by the iSLN.

We previously reported the details of the original ACSS-C2 method [8]. Preoperative assessment of lateral cervical radiographs is essential. The relationship between the mandible and C2–3 disk height was evaluated. If the mandible was lower than the C2–3 disk height in the extended position, the anterior approach would not be suitable. In such cases, the posterior approach should be considered. Nasal intubation is preferred to oral intubation because it allows full jaw closure to maximize access to C2 (Fig. 1). Patients were placed in the supine position with a shoulder roll to facilitate cervical extension and elevation of the mandible. A 5–6-cm wide transverse incision was made at the level of the hyoid bone, slightly to the left of the midline of the neck. The skin and platysma were incised horizontally followed by full longitudinal release of the inferior fascia. The submandibular gland, hyoid bone, and omohyoid and sternohyoid muscles, which were all located beneath the platysma, were identified (Fig. 2a). The submandibular gland was bluntly dissected and retracted cranially. The omohyoid and sternohyoid muscles, which are part of the infrahyoid muscles, were detached from the inferior edge of the hyoid bone and reflected caudally. The thyrohyoid membrane between the hyoid bone and thyroid cartilage was exposed deep to the infrahyoid muscles (Fig. 2b). Subsequently, surgical access to the vertebrae was performed in a plane along the lateral edge of the thyrohyoid membrane. Infrahyoid muscle detachment improved laryngeal mobility and helped in the identification of the iSLN, which traverses the C3–4 level and pierces the thyrohyoid membrane (Fig. 2c). Superior laryngeal vessels, which usually run along the iSLN, should be ligated. Additionally, the hypoglossal nerve courses at the level of the submandibular triangle toward the tongue from the carotid sheath; dissecting this nerve is not necessary as it runs more cephalad to the C2–3 disk. The prevertebral fascia was carefully incised while undermining the longus colli. Surgical exposure was facilitated by placing two self-retaining blade retractors beneath the longus colli muscle on both sides. In one- or two-level fusion, blade retractors were placed inferior to the hypoglossal nerve and superior to the iSLN. In cases of more than three-level fusion, both the hypoglossal nerve and iSLN were retracted superior to the blade retractors. Continuous retractor placement in the longitudinal direction should be avoided to prevent pressure-induced trauma to these neural structures. After adequate exposure of the vertebrae, subsequent corpectomy and/or discectomy was performed during the normal procedure (Fig. 2d). A tricortical autologous iliac bone graft and/or cage was fixed with an anterior cervical plate system (Fig. 2e). We repaired the omohyoid and sternohyoid muscle attachments to the hyoid bone using sutures before wound closure. To avoid injuries to the lingual artery and hypoglossal nerve that courses at the level of the submandibular triangle, suturing these muscles should be performed just above the hyoid bone (Fig. 2f). Figure 3 shows the schematic images of the infrahyoid muscles and the operative field dissection.

In each case, the judgment of whether immediate postoperative endotracheal extubation was suitable was performed by an anesthesiologist using a cuff leak test. Postoperatively, the patients were positioned with the head elevated at 20°, and oxygen saturation and subjective symptoms were monitored carefully in the intensive care unit for 2 days. On postoperative day 1, all patients were encouraged to resume intake of water and oral medications. Patients who were able to drink without difficulty gradually resumed eating solid food the next day. Furthermore, a rigid cervical collar was maintained for 3 months postoperatively.

## Results

Twelve patients were enrolled in this study. The baseline and procedural characteristics of the patients are summarized in Table 1. The mean age of the patients was 63 years (range 20–84 years). The mean follow-up period was 39.8 months (range 12–90 months). Five patients were treated for degenerative pathologies, six patients for traumatic disease, and one patient for neoplastic metastasis. Two patients had previously undergone cervical laminoplasty. The fused levels were C2–3 in five cases, C2–4 in two, C2–5 in four, and C2–6 in one. In all patients, the iSLN was identified and successfully preserved intraoperatively, and wide adequate exposure, including visualization of C2, enabled precise decompression, iliac bone grafting, and instrumentation.

**Table 1:** Baseline and surgical procedural characteristics of 12 patients treated with ACSS to C2

Case	Gender	Age (year)	Diagnosis	Symptoms	Previous surgery	Fused level	Surgical procedure	Operative time (min)	EBL (mL)	Complications
1	F	54	Hangman’s fx	Neck pain		C2–3	ACDF	183	10	
2	F	65	Hangman’s fx	Neck pain		C2–3	ACDF	134	50	
3	F	84	C2/3 traumatic spondylolisthesis	Myelopathy		C2–3	ACDF	104	5	
4	M	52	OPLL	Myelopathy		C2–5	ACCF	293	100	
5	M	20	Hangman’s fx	Neck pain		C2–3	ACDF	147	10	
6	M	71	C3 Metastasis	Neck pain		C2–4	ACCF	204	50	
7	F	78	OPLL	Myelopathy	LP	C2–6	Hybrid	355	150	Dural tear, C5 palsy, dysphagia
8	M	69	Deformity	Myelopathy		C2–5	ACCF	220	100	
9	M	58	OPLL	Myelopathy		C2–5	ACCF	243	100	
10	M	82	Hangman’s fx C3 vertebral fx	Neck pain Dysphagia		C2–4	ACCF	169	10	Pharyngeal perforation, dysphagia
11	F	74	OPLL	Myelopathy	LP	C2–5	ACCF	229	15	Surgical site infection
12	M	49	Hangman's fx	Neck pain		C2–3	ACDF	113	50	

*ACSS*, anterior cervical spine surgery; *EBL*, estimated blood loss; *fx*, fracture; *OPLL*, ossification of the posterior longitudinal ligament; *LP*, laminoplasty; *ACDF*, anterior cervical discectomy and fusion; *ACCF*, anterior cervical corpectomy and fusion; Hybrid, combined ACDF and ACCF

### Complications

Surgery-related complications are shown in Table 1. Incidental intraoperative dural tear and retropharyngeal perforation occurred in cases 7 and 10, respectively. Postoperative C5 palsy also occurred in case 7, with paralysis subsiding within 6 months after surgery. Ten of twelve patients were able to resume eating any solid food from postoperative day 2 or 3. Distinct dysphagia requiring temporary nasogastric tube feeding was observed in two patients (cases 7 and 10). In both patients, postoperative laryngeal fiberscope examination showed laryngeal mucosal edema and pooling of saliva and sputum around the glottis with no remarkable laterality. Thus, palsy of the iSLN causing left-side (surgical approach side) dominant unilateral paresthesia of the laryngeal mucosa was not considered. Meanwhile, swallowing improved gradually and resolved 2 weeks and 2 months postoperatively, respectively. In all patients, successful extubation was performed immediately after surgery. None of the patients experienced dyspnea, upper airway obstruction requiring reintubation, or postoperative hoarseness. One patient (case 11) required revision surgery for surgical site infection.

### Fusion outcomes

Bony fusion was achieved in all patients. None of the patients required revision owing to instrument failure or graft dislodgement.

### Illustrative cases

#### Case 10

**Fig. 4:**
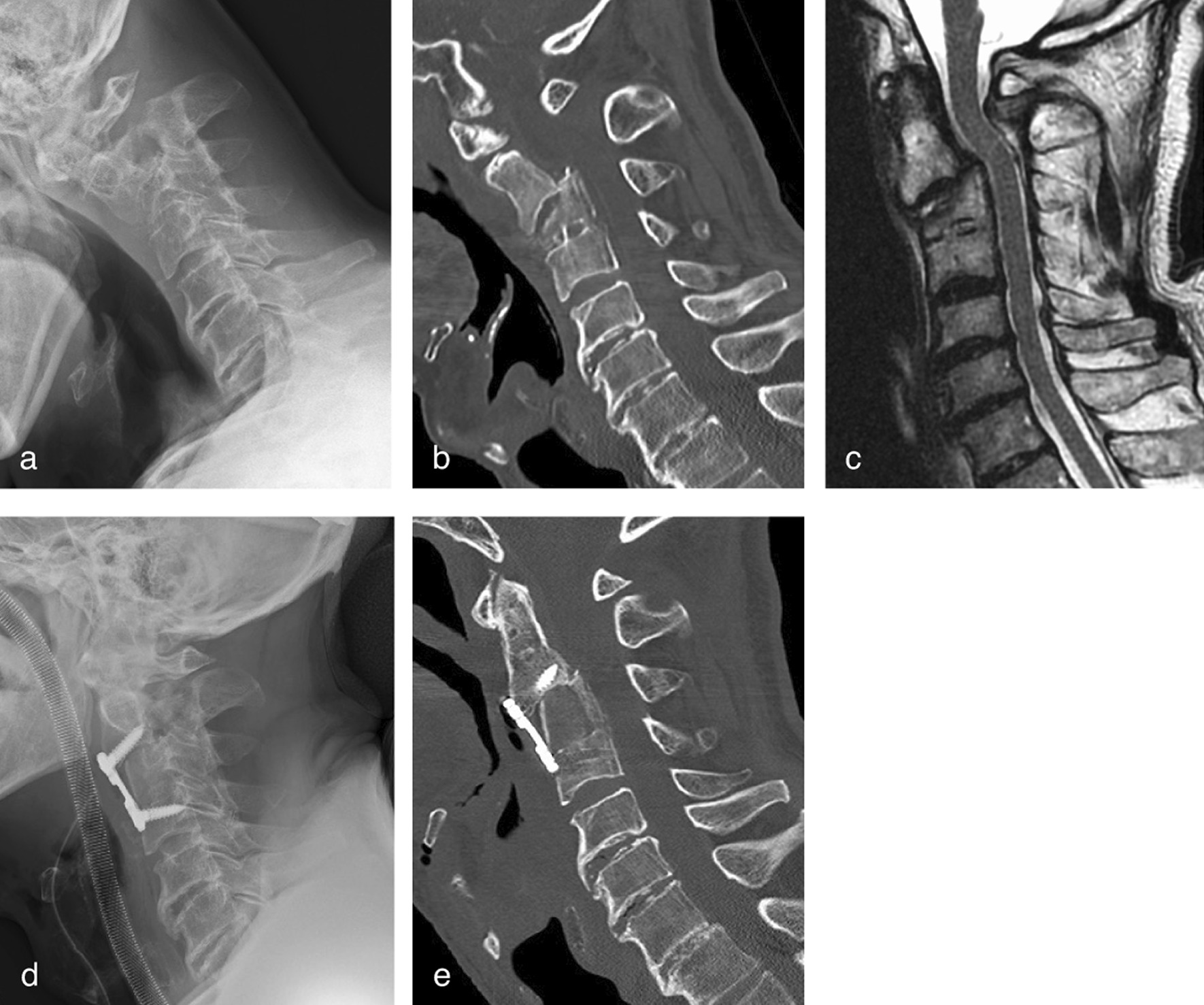
Case 10. **a** Preoperative lateral plain radiographs and **b** computed tomography sagittal images showing severe kyphotic deformity with C2 anterolisthesis and C3 vertebral collapse. **c** T2-weighed magnetic resonance sagittal image showing sigmoid course of the spinal cord without compressive lesion. **d** Postoperative lateral plain radiograph and **e** computed tomography sagittal image showing precise iliac bone grafting and C2–4 plate fixation

An 82-year-old man was diagnosed with Hangman’s fracture and C3 vertebral fracture and was referred to our hospital after a 1-month history of neck pain and gradual development of dysphagia. Preoperative images indicated severe C2–4 kyphotic deformity due to C2 anterolisthesis and C3 vertebral collapse (Fig. 4). Manual reduction and temporary halo vest immobilization were performed 1 week before surgery. He then underwent C2–4 anterior cervical corpectomy and fusion. Intraoperative preservation of the iSLN was successful; however, incidental pharyngeal wall perforation occurred due to post-traumatic adhesion around the pharynx, resulting in postoperative dysphagia. His symptoms gradually subsided within 2 months postoperatively.

#### Case 11

**Fig. 5:**
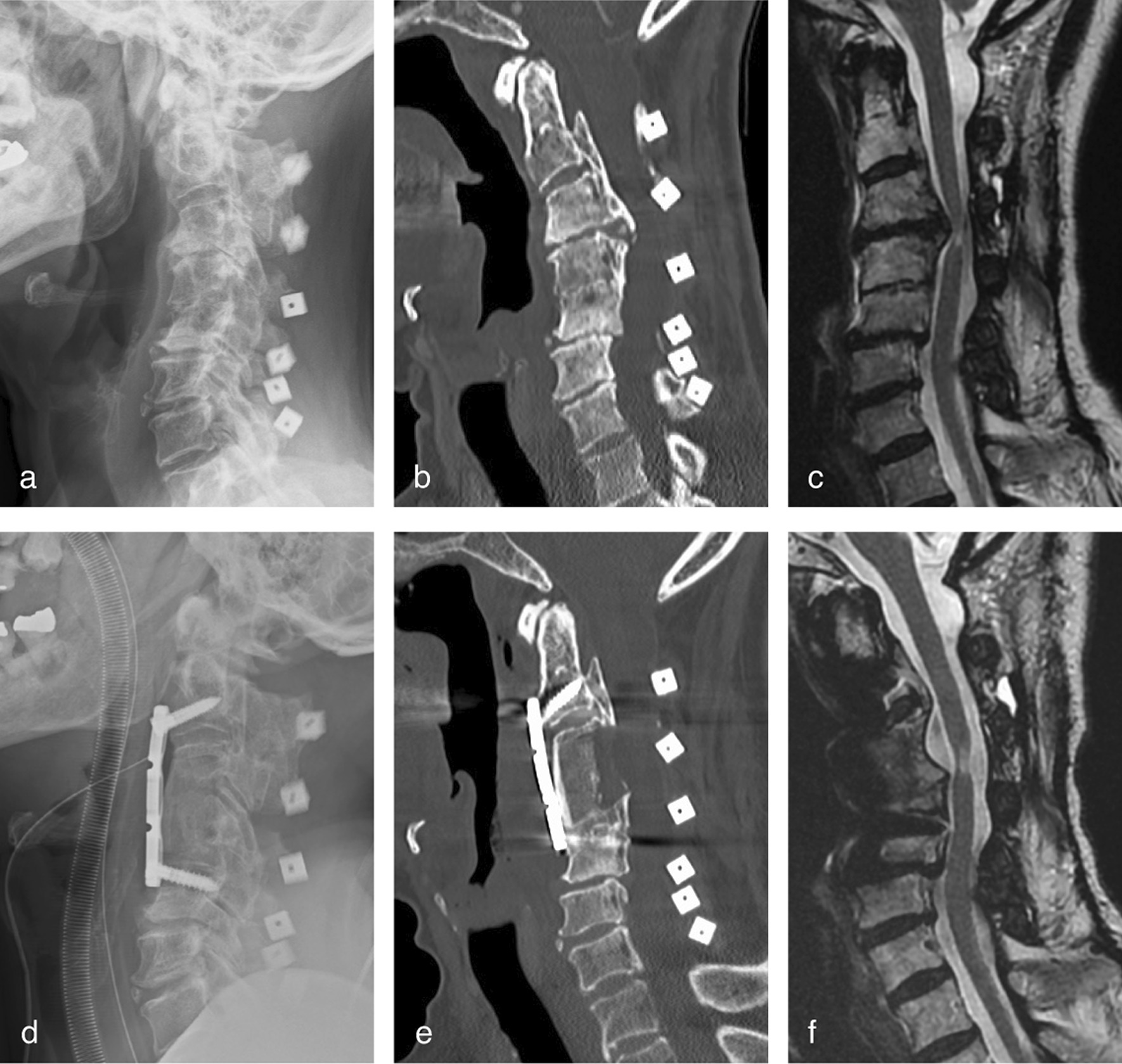
Case 11. **a** Preoperative lateral plain radiograph and **b** computed tomography sagittal image showing C2–5 continuous type ossification of the posterior longitudinal ligament (OPLL) and kyphotic deformity after laminoplasty. **c** Preoperative T2-weighed magnetic resonance sagittal image showing stenotic lesion with increased signal intensity of the spinal cord. **d** Postoperative lateral plain radiograph and **e** computed tomography sagittal image showing precise OPLL resection, iliac bone grafting, and C2–5 plate fixation. **f** Postoperative T2-weighed magnetic resonance sagittal image showing well-decompressed spinal cord

A 74-year-old woman who underwent laminoplasty for cervical ossification of the posterior longitudinal ligament (OPLL) 7 years earlier experienced recurrent myelopathy for 1 year. Preoperative imaging indicated OPLL progression with loss of cervical lordosis (Fig. 5). She underwent C2–5 anterior cervical corpectomy and fusion. Intraoperative preservation of the iSLN was successful, and precise decompression and instrumentation were performed. There was marked recovery from myelopathy without postoperative airway compliance or swallowing difficulty; however, deep surgical site infection occurred 2 months after surgery. She was treated with one-time debridement and postoperative antibiotic therapy. During revision surgery, the omohyoid and sternohyoid muscles, which were reattached to the hyoid bone in the initial surgery, were well-repaired without dehiscence or remarkable atrophic changes. The lateral border of the repaired omohyoid muscle is a good indicator of access to the retropharyngeal space.

## Discussion

Owing to the anatomical challenges of performing ACSS-C2, this procedure carries high risks of postoperative dysphagia and dyspnea. In this study, we described our modified approach for ACSS-C2 with temporary infrahyoid muscle detachment from the hyoid bone and investigated its surgical outcomes and complications in a case series of 12 patients. Adequate exposure of C2 and preservation of the iSLN were achieved in all cases. None of the patients experienced upper airway obstruction or underwent revision surgery due to instrumentation failure or graft dislodgement; however, two patients experienced transient dysphagia.

In ACSS-C2, the anatomical landmark of the skin incision is the hyoid bone. On the upper cervical levels, the mobility of the laryngopharynx and hyoid bone is more restricted by the supra- and infrahyoid muscles attached to the hyoid bone as compared with the mid-lower cervical levels. During anterior access to C2, the cervical spine must be extended to reduce mandibular interference. However, neck extension elevates the location of the hyoid bone, increasing infrahyoid muscle tightness. Therefore, to adequately expose C2, forceful medial retraction of the laryngopharynx, including the infrahyoid muscles, is required. However, pressure-induced trauma of neural structures, connective tissues, and muscle fibers can cause severe retropharyngeal edema, which may lead to postoperative dysphagia.

Several other anatomical features at the upper cervical level may be responsible for the high complication rates of ACSS-C2. First, the oropharynx, which is a relatively soft tissue without skeletal protection [6], is located at the C2–3 to C3–4 levels. Thus, when C2 is exposed, retropharyngeal edema, physical airway stenosis, and consequent respiratory failure is more likely to occur [3, 7]. Second, the iSLN, which courses at the C3–4 level and pierces the thyrohyoid membrane, is more likely to be injured when approaching the upper cervical levels [5, 9]. iSLN palsy is one of the reasons for the higher incidence of postoperative dysphagia in upper cervical level surgery [10, 11]. To decrease the risk of dysphagia, it is essential to have adequate anatomical knowledge of the iSLN and to correctly identify it during surgical field dissection [1, 12]. However, infrahyoid muscles overlapping the thyrohyoid membrane may sometimes make it difficult to identify the iSLN.

In our modified approach, we temporarily detached the omohyoid and sternohyoid muscles from the hyoid bone to improve mobility of the laryngopharynx, including the hyoid bone. Infrahyoid muscle detachment can provide better accessibility to C2 without forceful medial retraction of the laryngopharynx and can reduce postoperative retropharyngeal edema. Furthermore, this procedure can reveal the thyrohyoid membrane, which is the piercing point of the iSLN, enabling the easy identification and preservation of the iSLN. Therefore, we believe that our modified approach with temporary infrahyoid muscle detachment can reduce the risk of postoperative dysphagia and dyspnea after ACSS-C2 compared to the traditional approach.

In otolaryngology, infrahyoid myotomy, which is usually performed in combination with other procedures, reportedly improves swallowing [13]. The infrahyoid consists of four muscles: the omohyoid, sternohyoid, sternothyroid, and thyrohyoid; these muscles are responsible for positioning the hyoid bone along with the suprahyoid muscles (Fig. 3a). All infrahyoid muscles, except for the thyrohyoid muscle, depress the hyoid bone. Infrahyoid myotomy can assist in elevating the hyoid bone and larynx during the swallowing phase. Hence, infrahyoid muscle detachment from the hyoid bone would not negatively affect swallowing function. Repairing the infrahyoid muscle attachment on the hyoid bone may not be essential; however, we recommend doing so. If revision surgery is required, the lateral border of the repaired omohyoid muscle would be a useful landmark for accessing the retropharyngeal space.

To the best of our knowledge, previous case series studies of ACSS-C2, including multi-level fusion cases, have shown that the incidence of dysphagia is 21.4–77.8% [3, 14–19], although the operational definitions of dysphagia varied in each study. Of these studies, two case series included cases of persistent dysphagia (symptoms were prolonged until the final follow-up) [3, 16] and cases of dyspnea that required unplanned reintubation. The authors reported that the incidence of reintubation was 14.4% (3/16 cases), which was significantly higher than that of lower-level fusion cases performed during the same period [3]. In this study, 16.7% (2/12 cases) of patients experienced transient dysphagia, which is relatively low compared to that in previous studies. Additionally, none of our patients had persistent dysphagia or upper airway obstruction, suggesting that our technique is relatively safe.

Both patients who experienced distinct dysphagia (cases 7 and 10) were older adults (78 years and 81 years, respectively) and underwent multi-level corpectomy and fusion. A cadaveric study demonstrated that the developing space between the hypoglossal nerve and the iSLN may sufficiently expose C2–3; however, an approach to C2–3 from a more caudal level may not be performed without injuring the iSLN, which traverses the C3–4 level [20]. Our approach was advantageous in facilitating the identification of the iSLN, thus avoiding incidental ligation. In contrast, a limitation of our approach was the unavoidable stretching of the iSLN, which may result in injury when retracted cranially or caudally in multi-level fusion cases.

From a pathophysiological perspective, older patients are more likely to develop dysphagia due to weak oropharyngeal muscles and impaired pharyngeal sensitivity secondary to the normal aging process [21, 22]. Several studies have demonstrated that older age is a significant predictor of postoperative dysphagia following ACSS [23–25]. In this study, the low tolerance of neural, muscular, and mucosal tissue against the pressure of blade retractors in older patients could have also contributed to the development of dysphagia. Therefore, we do not recommend multi-level ACSS-C2 in older patients, and alternative procedures, such as posterior decompression and fusion, should be considered instead.

This study has some limitations. First, the number of patients was small because the surgical indications for ACSS-C2 were limited. The incidence of dyspnea requiring reintubation, which is relatively rare, may have been underestimated. Second, we could not compare surgical outcomes and complications between cases with and without infrahyoid muscle detachment. Third, we could not perform a quantitative analysis on how pressure to the pharynx is reduced via infrahyoid muscle detachment during medial retraction in animal or cadaveric experiments. Further studies with more cases are required to determine the surgical value of our technique and the incidence of dysphagia and dyspnea after ACSS-C2.

## Conclusions

Our modified approach to ACSS-C2 with temporary infrahyoid muscle detachment from the hyoid bone may improve laryngeal mobility and facilitate easy identification of the iSLN, subsequently decreasing the risk of postoperative persistent dysphagia and dyspnea. Multi-level fusion in older patients should be avoided because of the high risk of postoperative dysphagia; hence, alternative procedures should be considered.

## Data Availability

The datasets generated during and/or analyzed during the current study are available from the corresponding author on reasonable request.

## Abbreviations

ACSS-C2: Anterior cervical spine surgery to C2
iSLN: Internal branch of the superior laryngeal nerve
OPLL: Ossification of the posterior longitudinal ligament
